# T1 and T2 mapping cardiovascular magnetic resonance to monitor the course of myocarditis

**DOI:** 10.1186/1532-429X-18-S1-O99

**Published:** 2016-01-27

**Authors:** Ulf K Radunski, Sebastian Bohnen, Gunnar Lund, Lena M Wilmink, Yana Looft, Mirac Senel, Christian Stehning, Bernhard Schnackenburg, Gerhard Adam, Stefan Blankenberg, Kai Muellerleile

**Affiliations:** 1 grid.13648.380000000121803484Cardiology, University Heart Center, Hamburg, Germany; 2 grid.13648.380000000121803484Radiology, University Medical Center, Hamburg, Germany; 3 Philips Research Germany, Hamburg, Germany; 4 Philips Healthcare Germany, Hamburg, Germany

## Background

Myocarditis is a heterogeneous disease with a large spectrum of possible clinical courses ranging from complete healing to end stage heart failure. However, there is a lack of reliable prognostic factors to identify patients with an adverse outcome. T1 and T2 mapping are promising novel diagnostic cardiovascular magnetic resonance (CMR) tools for a quantitative assessment of the dynamic tissue changes such as edema, necrosis and fibrosis during the course of myocarditis. Thus, we evaluated T1 and T2 mapping CMR for the evaluation of disease activity in patients with myocarditis.

## Methods

This study included 26 patients with myocarditis, who underwent CMR at 1.5 Tesla acute at baseline (BL) and at 3 months follow-up (FU) after the initial presentation with myocarditis, respectively. T1 and T2 mapping CMR were performed in addition to a conventional CMR protocol in myocarditis. T1 quantification was performed on three short axis using the modified Look-Locker inversion-recovery (MOLLI) sequence before and 15 minutes after administration of 0.075 mmol/kg Gadolinium-BOPTA. T2 quantification was performed using a free-breathing, navigator-gated multi-echo sequence. Maps were generated using a dedicated plug-in for the OsiriX software to calculate global myocardial T2, native/post-contrast T1 and extracellular-volume-fraction (ECV).

## Results

Left ventricular ejection fraction improved from 49 (29-62) % at BL to 54 (42-61) % at FU (p < 0.001). Global myocardial T2 significantly decreased from 60 (58-63) ms at BL to 58 (55-61) ms at FU (p < 0.05). Similarly, global myocardial native T1 decreased from 1119 (1097-1149) ms to 1075 (1037-1113) ms (p < 0.01). Furthermore, global myocardial ECV was significantly lower at FU with 26 (25-29) % compared to 28 (26-31) % at BL (p < 0.05). There were no significant differences between global myocardial T2, native T1 and ECV values of patients with myocarditis at FU and healthy controls.

## Conclusions

T1 and T2 mapping CMR techniques offer novel insights into the course of myocarditis. Our findings suggest that mapping techniques are useful for the assessment of disease activity and may serve as prognostic factors for patients with myocarditis.

